# Fulvestrant 500 mg vs 250 mg in postmenopausal women with estrogen receptor-positive advanced breast cancer: a randomized, double-blind registrational trial in China

**DOI:** 10.18632/oncotarget.10254

**Published:** 2016-06-23

**Authors:** Qingyuan Zhang, Zhimin Shao, Kunwei Shen, Li Li, Jifeng Feng, Zhongsheng Tong, Kangsheng Gu, Xiaojia Wang, Binghe Xu, Guofang Sun, Huifang Chen, Yuri Rukazenkov, Zefei Jiang

**Affiliations:** ^1^ Harbin Medical University Cancer Hospital, Harbin, Heilongjiang, China; ^2^ Fudan University Shanghai Cancer Center, Shanghai, China; ^3^ Shanghai Ruijin Hospital, Shanghai, China; ^4^ The First Affiliated Hospital of Dalian Medical University, Dalian, Liaoning, China; ^5^ Jiangsu Cancer Hospital, Nanjing, Jiangsu, China; ^6^ Tianjin Cancer Hospital, Tianjin, China; ^7^ The First Affiliated Hospital of Anhui Medical University, Hefei, Anhui, China; ^8^ Zhejiang Cancer Hospital, Hangzhou, Zhejiang, China; ^9^ Cancer Hospital and Institute, Chinese Academy of Medical Sciences and Peking Union Medical College, Beijing, China; ^10^ AstraZeneca, Shanghai, China; ^11^ AstraZeneca, Macclesfield, UK; ^12^ 307 Hospital of Chinese People's Liberation Army, Beijing, China

**Keywords:** advanced breast cancer, fulvestrant, endocrine therapy, hormone receptor-positive breast cancer

## Abstract

The international CONFIRM study showed that fulvestrant 500 mg improved progression-free survival (PFS) vs fulvestrant 250 mg in postmenopausal women with estrogen receptor (ER)-positive locally advanced/metastatic breast cancer (LA/MBC). In this randomized, double-blind study, postmenopausal Chinese women with ER-positive LA/MBC and progression after endocrine therapy received fulvestrant 500 mg (days 0, 14, 28, and every 28 days thereafter) or fulvestrant 250 mg (every 28 days). Consistency with the international study was assumed if the hazard ratio (HR) for comparison of PFS (primary endpoint) was < 1 (stratified log-rank test). The study was not powered to assess between-group differences.

In total, 221 patients were randomized (fulvestrant 500 mg: *n* = 111; fulvestrant 250 mg: *n* = 110). Baseline characteristics were balanced. Median PFS was 8.0 months with fulvestrant 500 mg vs 4.0 months with 250 mg (HR = 0.75; 95% confidence interval [CI] 0.54−1.03; *P* = 0.078). PFS (HR; 95% CI) favored fulvestrant 500 mg in post-antiestrogen (0.86; 0.54−1.37) and post-aromatase inhibitor (0.65; 0.42−1.03) settings. No new safety considerations were observed. These results are consistent with the international CONFIRM study, supporting the superior clinical benefit of fulvestrant 500 mg in women with ER-positive LA/MBC experiencing progression following prior endocrine therapy.

## INTRODUCTION

Breast cancer is one of the most common female cancers, with approximately 521,900 women dying of breast cancer annually [1]. Endocrine therapies are an established treatment for postmenopausal women with hormone receptor-positive advanced breast cancer (estrogen receptor [ER]-positive and/or progesterone receptor [PgR]-positive) [2, 3]. Given that many patients eventually experience disease progression in this setting, non-cross-resistant endocrine therapies are required to provide optimal disease control throughout the treatment cascade [4].

Fulvestrant is a selective ER degrader that binds to and blocks the ER, and increases ER degradation [5]. Fulvestrant was originally approved at a dose of 250 mg/month following global registration studies that demonstrated that fulvestrant 250 mg was at least as effective as anastrozole for the treatment of advanced breast cancer in postmenopausal women with disease progression following prior endocrine therapy [6, 7]. Furthermore, fulvestrant has been shown to induce a response in tumors which have developed resistance to antiestrogen and aromatase inhibitor therapies [4, 8–10].

In the international phase III COmparisoN of Faslodex In Recurrent or Metastatic breast cancer (CONFIRM) study (NCT00099437), a fulvestrant 500 mg dose regimen (fulvestrant 500 mg/month with an additional 500 mg dose on day 14 of month 1) was associated with significantly longer progression-free survival (PFS) compared with the 250 mg dose (median PFS: 6.5 months vs 5.5 months; hazard ratio [HR] = 0.80; 95% confidence interval [CI] 0.68−0.94; *P* = 0.006) without increasing the incidence or severity of adverse events (AEs) [11]. In a follow-up analysis, fulvestrant 500 mg was associated with a clinically significant 4.1-month improvement in median overall survival (OS) vs fulvestrant 250 mg (median OS: 26.4 months vs 22.3 months, respectively; HR = 0.81; 95% CI 0.69−0.96; nominal *P* = 0.02) [12].

In China, a registration trial confirmed that fulvestrant 250 mg is effective in postmenopausal women, which led to its approval for the treatment of postmenopausal women with ER-positive locally advanced or metastatic breast cancer and disease relapse during or after adjuvant antiestrogen therapy or disease progression during antiestrogen therapy [13]. The current study was therefore designed to compare the efficacy and safety of fulvestrant 500 mg vs 250 mg in a Chinese population.

## RESULTS

### Patients

**Figure 1:**
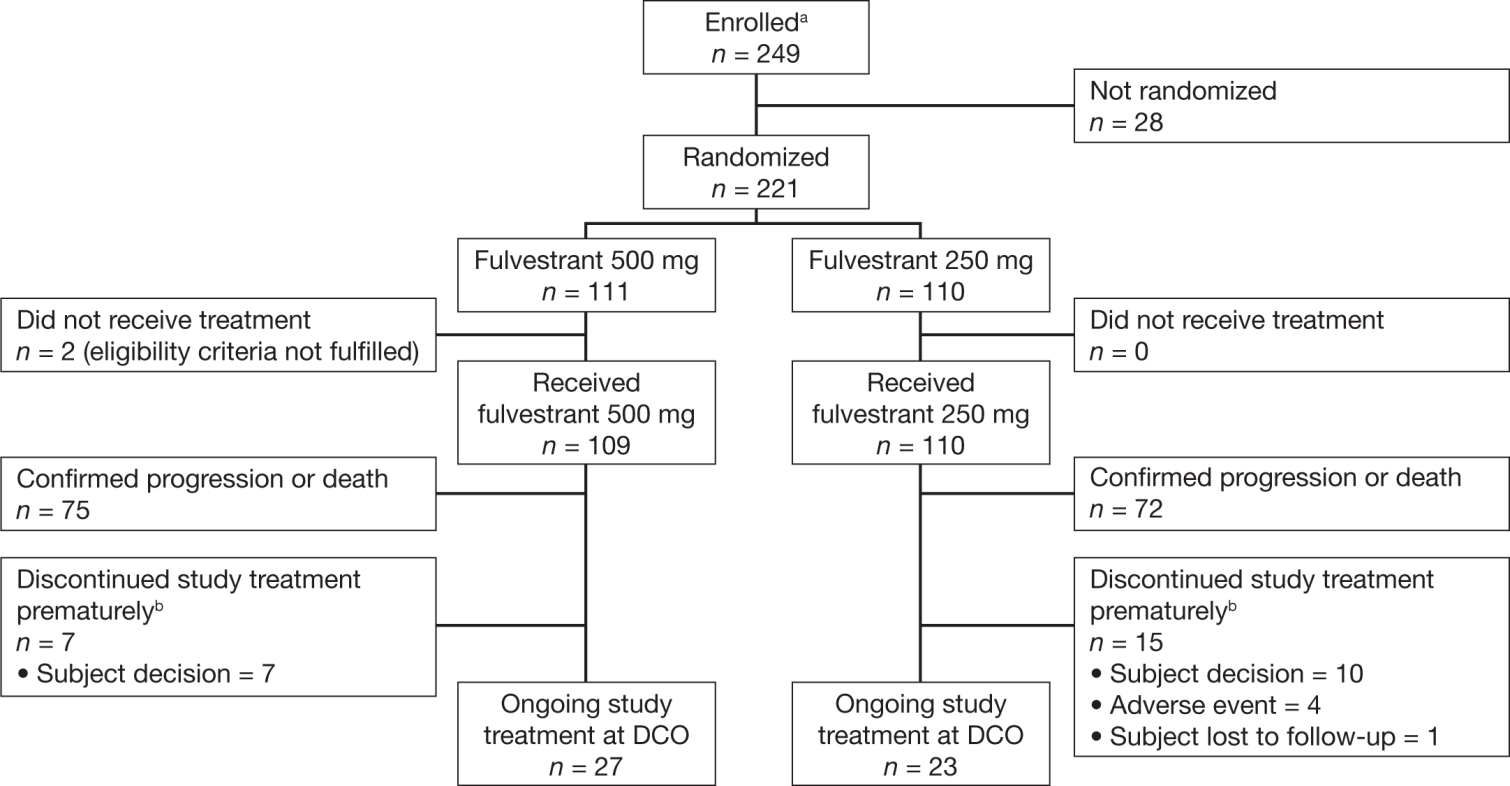
Patient disposition (full analysis set) ^a^Informed consent received. ^b^Patients who discontinued treatment prematurely due to reasons other than confirmed disease progression or death. DCO, data cut-off.

Patients were randomized at 23 centers in China between March 9, 2011 and December 30, 2013. The data cut-off for this analysis was March 25, 2014, at which time 152 disease progression events had occurred. Of 221 patients enrolled, 111 were randomized to fulvestrant 500 mg and 110 were randomized to fulvestrant 250 mg (full analysis set). Two patients in the fulvestrant 500 mg group did not receive treatment and were therefore excluded from the safety analysis set (Figure 1).

The evaluable-for-response analysis set included 57 patients in the fulvestrant 500 mg group and 66 patients in the 250 mg group with ≥ 1 target lesion at baseline. A total of 121 patients (55%) had received antiestrogen therapy as their last endocrine therapy prior to randomization, while 100 (45%) had received aromatase inhibitor therapy (Table 1). Demographic and baseline characteristics were balanced between the fulvestrant 500 mg and 250 mg groups (Table 1), and were consistent with those in the international CONFIRM study [11].

**Table 1:** Demographic and baseline characteristics (full analysis set)

	Fulvestrant 500 mg (*n* = 111)	Fulvestrant 250 mg (*n* = 110)	Total (*n* = 221)
Sex, *n* (%)			
Female	111 (100)	110 (100)	221 (100)
Age (years)			
Mean (SD)	53.6 (10.1)	53.1 (10.2)	53.3 (10.2)
Median (range)	55 (26−80)	55 (31−76)	55 (26−80)
Age group, *n* (%)			
< 50 years	37 (33.3)	40 (36.4)	77 (34.8)
50−< 65 years	61 (55.0)	56 (50.9)	117 (52.9)
≥ 65 years	13 (11.7)	14 (12.7)	27 (12.2)
Weight (kg)			
Mean (SD)	61.0 (8.5)	60.5 (10.0)	60.7 (9.3)
Median (range)	60.0 (35.0−85.0)	58.8 (42.0−88.0)	60.0 (35.0−88.0)
BMI (kg/m^2^)			
Mean (SD)	24.0 (3.2)	23.9 (3.7)	23.9 (3.5)
Median (range)	23.7 (14.4−34.0)	23.1 (16.3−35.1)	23.4 (14.4−35.1)
BMI (kg/m^2^) group, *n* (%)			
Underweight (< 18.5)	2 (1.8)	2 (1.8)	4 (1.8)
Normal (≥ 18.5−< 24)	56 (50.5)	64 (58.2)	120 (54.3)
Overweight (≥ 24−< 28)	45 (40.5)	25 (22.7)	70 (31.7)
Obese (≥ 28)	8 (7.2)	19 (17.3)	27 (12.2)
Height (cm)			
Mean (SD)	159.4 (4.5)	159.1 (5.5)	159.2 (5.0)
Median (range)	160 (150−170)	160 (146−172)	160 (146−172)
Prior endocrine therapy, *n* (%)			
Adjuvant endocrine therapy	108 (97.3)	103 (93.6)	211 (95.5)
Antiestrogen	58 (52.3)	61 (55.5)	119 (53.8)
Aromatase inhibitor	50 (45.0)	42 (38.2)	92 (41.6)
Endocrine therapy for advanced disease	35 (31.5)	30 (27.3)	65 (29.4)
Antiestrogen	7 (6.3)	7 (6.4)	14 (6.3)
Aromatase inhibitor	28 (25.2)	23 (20.9)	51 (23.1)
Last endocrine therapy prior to randomization, *n* (%)			
Antiestrogen	58 (52.3)	63 (57.3)	121 (54.8)
Aromatase inhibitor	53 (47.7)	47 (42.7)	100 (45.2)
Prior chemotherapy,^a^ *n* (%)			
Adjuvant chemotherapy	98 (88.3)	94 (85.5)	192 (86.9)
Chemotherapy for advanced disease	25 (22.5)	20 (18.2)	45 (20.4)
Prior radiotherapy, *n* (%)			
Adjuvant	55 (49.5)	53 (48.2)	108 (48.9)
Palliative	11 (9.9)	12 (10.9)	23 (10.4)

a Patients may appear under more than one previous treatment modality.

BMI, body mass index; SD, standard deviation.

### Efficacy

**Figure 2:**
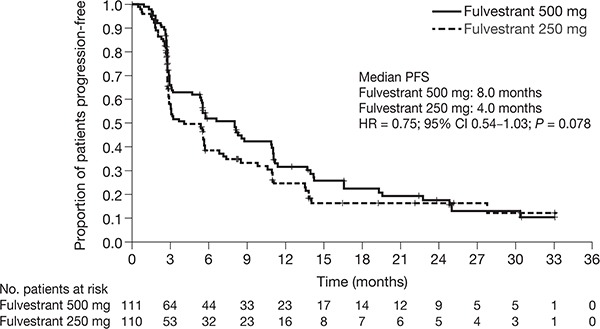
Kaplan-Meier analysis of PFS with fulvestrant 500 mg vs fulvestrant 250 mg (full analysis set) CI, confidence interval; HR, hazard ratio; PFS, progression-free survival.

A similar proportion of patients experienced a progression event in the fulvestrant 500 mg and 250 mg groups (68% [76/111] vs 69% [76/110], respectively). The median (95% CI) PFS was 8.0 (5.5–10.9) months in the fulvestrant 500 mg group vs 4.0 (2.9–5.7) months in the 250 mg group (HR = 0.75; 95% CI 0.54−1.03; *P* = 0.078) (Figure 2). At 12 months, 32% and 25% of patients were progression-free in the fulvestrant 500 mg and 250 mg groups, respectively; these figures were 18% and 17%, respectively, at 24 months. The study was not powered for statistical significance.

**Figure 3:**
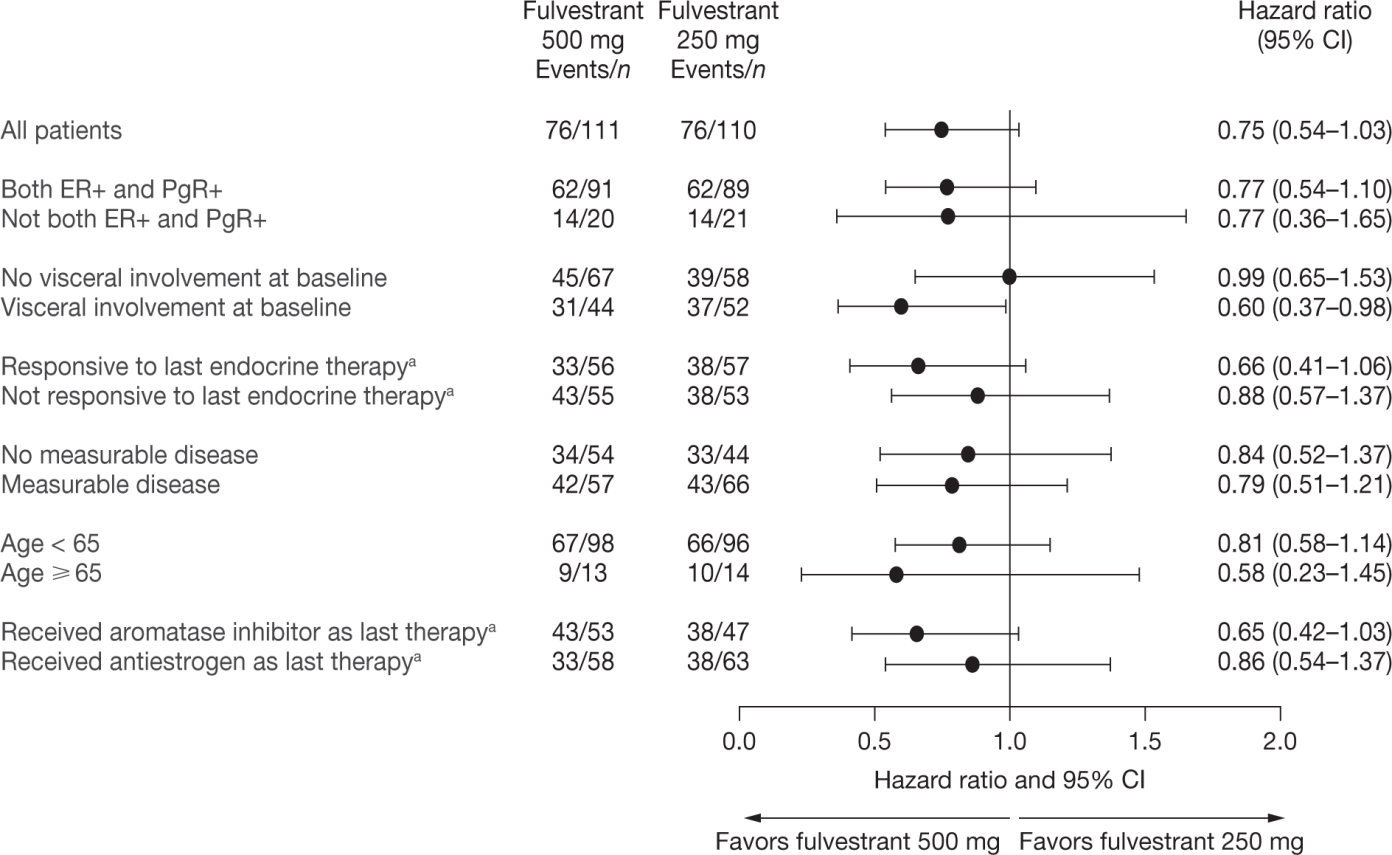
Subgroup analysis of PFS (full analysis set) ^a^Prior to study enrollment. CI, confidence interval; ER, estrogen receptor; PFS, progression-free survival; PgR, progesterone receptor.

Subgroup analyses were consistent with the overall effect on PFS (Figure 3). At data cut-off, more progression events had occurred in the post-aromatase inhibitor subgroup than the post-antiestrogen subgroup (81% [81/100] vs 59% [71/121], respectively). The median PFS in the post-antiestrogen subgroup was 8.1 months vs 5.6 months in the fulvestrant 500 mg and 250 mg groups, respectively (HR = 0.86; 95% CI 0.54−1.37) (Supplementary Figure S1A). In the post-aromatase inhibitor group, median PFS was 5.8 months vs 2.9 months in the fulvestrant 500 mg and 250 mg groups, respectively (HR = 0.65; 95% CI 0.42−1.03) (Supplementary Figure S1B).

In patients with target lesions at baseline, the objective response rate (ORR) was 28% (16/57) in the fulvestrant 500 mg group and 17% (11/66) in the 250 mg group (odds ratio = 1.44; 95% CI 0.93–2.44; *P* = 0.107) (Table 2). In patients with an objective response, the median duration of response (DoR) from onset of response was 16.6 months in the fulvestrant 500 mg group and 22.2 months in the 250 mg group. The clinical benefit rate (CBR) was 48% (53/111) in the fulvestrant 500 mg group and 33% (36/110) in the 250 mg group (full analysis set) (odds ratio = 1.37; 95% CI 1.04–1.80; *P* = 0.023). In patients achieving clinical benefit, the median duration of clinical benefit (DoCB) was 14.3 months in the fulvestrant 500 mg group and 13.8 months in the fulvestrant 250 mg group.

**Table 2:** Summary of objective response and clinical benefit (full analysis set)

	Fulvestrant 500 mg (*n* = 111)	Fulvestrant 250 mg (*n* = 110)
Complete response, *n* (%)	2 (1.8)	2 (1.8)
Partial response, *n* (%)	14 (12.6)	10 (9.1)
Objective response,^a^ *n* (%)	16 (14.4)	12 (10.9)
Stable disease ≥ 24 weeks, *n* (%)	37 (33.3)	24 (21.8)
Clinical benefit, *n* (%)	53 (47.7)	36 (32.7)
Stable disease < 24 weeks, *n* (%)	18 (16.2)	21 (19.1)
Progression,^b^ *n* (%)	37 (33.3)	48 (43.6)
Not evaluable, *n* (%)	4 (3.6)	6 (5.5)

a Complete response plus partial response rate in patients with measurable disease was 28.1% (16 of 57 patients) with fulvestrant 500 mg and 16.7% (11 of 66 patients) with fulvestrant 250 mg.

b Includes one death in the fulvestrant 250 mg group.

### Safety

Median duration of exposure was 6.5 months in the fulvestrant 500 mg group and 3.8 months in the 250 mg group. The number of patients who experienced AEs was similar between fulvestrant 500 mg and 250 mg treatment groups (68 [62.4%] vs 65 [59.1%]) (Table 3). Serious AEs were reported for 13 patients (6%). One patient in the fulvestrant 250 mg group reported two serious AEs that were considered by the investigator to be treatment-related (anemia and decreased platelet count [both grade 3]) and three non-treatment-related serious AEs (pulmonary infection, cardiac failure and dyspnea [all grade 2]), and discontinued treatment; all SAEs for this patient began 49–55 days after treatment initiation. Three other patients discontinued treatment due to a non-treatment-related AE (hypoglycemia [grade 3; occurring 51 days after treatment initiation]; erythematous skin rash and painful subcutaneous nodules in the arm [both grade 1; both occurring 15 days after treatment initiation]; and pulmonary infection [grade 4; occurring 1 day after treatment initiation], respectively) in the fulvestrant 250 mg group. There were no discontinuations due to an AE in the fulvestrant 500 mg group. Six patients, three in each treatment group, died due to disease progression during the study.

**Table 3:** Most common (frequency of ≥ 5%) adverse events with fulvestrant 500 mg and fulvestrant 250 mg (safety analysis set)

	Fulvestrant 500 mg (*n* = 109)	Fulvestrant 250 mg (*n* = 110)	Total (*n* = 219)
Patients with any AEs, *n* (%)	68 (62.4)	65 (59.1)	133 (60.7)
Injection-site reaction, *n* (%)	12 (11.0)	13 (11.8)	25 (11.4)
Injection-site pain, *n* (%)	8 (7.3)	10 (9.1)	18 (8.2)
Pyrexia, *n* (%)	11 (10.1)	6 (5.5)	17 (7.8)
Fatigue, *n* (%)	9 (8.3)	7 (6.4)	16 (7.3)
Nausea, *n* (%)	6 (5.5)	7 (6.4)	13 (5.9)
Back pain, *n* (%)	8 (7.3)	4 (3.6)	12 (5.5)
Cough, *n* (%)	2 (1.8)	6 (5.5)	8 (3.7)

AE, adverse event.

## DISCUSSION

Data from the present study support the superior clinical benefit of fulvestrant 500 mg vs 250 mg in postmenopausal Chinese women with ER-positive locally advanced or metastatic breast cancer. The study was not powered to detect significant differences between treatment groups; results met the predefined criterion for consistency with the international CONFIRM study (HR < 1 for the treatment comparison of PFS).

Patients who received fulvestrant 500 mg had a longer PFS, a higher ORR, a higher CBR, and a longer DoCB than those who received 250 mg. However, DoR was numerically higher in the fulvestrant 250 mg group (fulvestrant 500 mg: 16.6 months; fulvestrant 250 mg: 22.2 months); this difference most likely reflects the small sample size (*n* = 16 and *n* = 11, respectively).

Efficacy observations in the fulvestrant 250 mg group appear comparable to those seen in a previous study [13]. However, PFS data for fulvestrant 250 mg in that study were lower than in the global registration studies for fulvestrant 250 mg [10], which may reflect the higher proportion of patients with prior chemotherapy, potentially indicating a worse prognosis for those patients.

HRs for PFS favoring fulvestrant 500 mg over 250 mg were observed in both the post-antiestrogen and post-aromatase inhibitor settings. The HRs in the post-antiestrogen and post-aromatase inhibitor subgroups in the international CONFIRM study also both favored fulvestrant 500 mg over 250 mg (HR = 0.76; 95% CI 0.62−0.94 and HR = 0.85; 95% CI 0.67−1.08, respectively). The later accrual of patients to the post-antiestrogen subgroup reflects the lower maturity for PFS in this subgroup compared with the post-antiestrogen subgroup in the international CONFIRM study (59% vs 83%).

Fulvestrant is recommended as one of the treatment choices for postmenopausal patients with hormone-sensitive (ER- and/or PgR-positive) advanced breast cancer by the most recent National Comprehensive Cancer Network Guidelines [16]. Aromatase inhibitor or fulvestrant is recommended as treatment choice for postmenopausal advanced breast cancer after tamoxifen treatment failure; steroidal aromatase inhibitor (with or without everolimus) or fulvestrant 500 mg is recommended as treatment choice for postmenopausal advanced breast cancer after non-steroidal aromatase inhibitor treatment failure by the most recent Chinese Breast Cancer Guidelines [17].

The patient population in the current study was broadly comparable to the international CONFIRM study and included a similar proportion of patients who had received previous antiestrogen or aromatase inhibitor treatment; however, patients tended to be younger in the current study. For example, only 12% of patients were aged ≥ 65 years compared with 40% in the international CONFIRM study. Furthermore, a higher proportion of patients in the present study had received prior adjuvant chemotherapy (86.9%) compared with the international CONFIRM study (52.3%), which may reflect different prescribing trends between participating countries. The safety profiles of fulvestrant 500 mg and 250 mg were similar, and consistent with the known safety profile of fulvestrant.

Recent clinical trials have shown that endocrine therapy in combination with targeted therapies can improve PFS compared with endocrine therapy alone. Data from the phase III BOLERO-2 trial, comparing everolimus and exemestane vs exemestane and placebo in patients with hormone receptor-positive, human epidermal growth factor receptor type 2 (HER2)-non-amplified advanced breast cancer who had recurrence or progression while receiving previous therapy with a non-steroidal aromatase inhibitor in the adjuvant setting or to treat advanced disease (or both), reported a marked PFS advantage for the combination; median PFS was 6.9 months with everolimus plus exemestane and 2.8 months with placebo plus exemestane, according to assessments by local investigators (HR for progression or death = 0.43; 95% CI 0.35–0.54; *P* < 0.001). Combination therapy was associated with a higher incidence of AEs than exemestane alone [14]. TAnDEM, a randomized phase III study comparing anastrozole with or without trastuzumab in postmenopausal women with HER2/hormone receptor-positive metastatic breast cancer (previous treatment with tamoxifen as adjuvant or hormonal therapy for metastatic breast cancer or anastrozole if begun up to 4 weeks before random assignment was permitted) indicated that the combination arm experienced significant improvements in PFS compared with patients receiving anastrozole alone (HR = 0.63; 95% CI 0.47–0.84; median PFS 4.8 vs 2.4 months; *P* = 0.0016), although AEs and serious AEs were more frequent with the combination [15]. Data from the phase III PALOMA-3 trial, comparing fulvestrant 500 mg plus palbociclib vs fulvestrant 500 mg alone in the second-line or subsequent setting in postmenopausal women (or pre- or perimenopausal women receiving goserelin) with advanced hormone receptor-positive, HER2-negative breast cancer that had relapsed or progressed during prior endocrine therapy, reported a marked PFS advantage for the combination; the median PFS was 9.2 months with palbociclib plus fulvestrant and 3.8 months with placebo plus fulvestrant (HR for disease progression or death = 0.42; 95% CI 0.32–0.56; *P* < 0.001). The median PFS for fulvestrant 500 mg alone was shorter in PALOMA-3 than in previous studies, indicative of the younger, higher-risk, and more heavily pretreated population recruited into the PALOMA-3 trial [18].

In conclusion, this study was consistent with the international phase III CONFIRM study and provides evidence for the efficacy of fulvestrant 500 mg in Chinese women with ER-positive advanced breast cancer whose disease had progressed or relapsed after prior endocrine therapy (antiestrogen or aromatase inhibitor).

## MATERIALS AND METHODS

### Ethics statement

This investigation was conducted in accordance with the Declaration of Helsinki and national and international guidelines, and has been approved by the authors' institutional review board. All patients provided informed, written consent. The trial was registered with ClinicalTrials.gov (NCT01300351).

### Study design and participants

This was a randomized, double-blind, multicenter study conducted in China. Eligible patients were postmenopausal women with ER-positive locally advanced or metastatic breast cancer with a World Health Organization performance status of 0−2, and had either measurable disease (as per RECIST 1.1 criteria) or non-measurable disease with bone lesions, lytic lesions, or mixed (lytic and sclerotic) lesions. Patients were required to have either: relapsed during, or within 12 months of completion of, adjuvant endocrine therapy (tamoxifen, toremifene, or aromatase inhibitor [treatments included anastrozole, letrozole, and exemestane]); progressed on endocrine therapy (tamoxifen, toremifene, or aromatase inhibitor), provided that this endocrine treatment was started at least 12 months after the completion of adjuvant endocrine treatment; or progressed on endocrine therapy (tamoxifen, toremifene, or aromatase inhibitor), given as first treatment for patients with *de novo* advanced breast cancer.

Patients were excluded if they had life-threatening metastatic visceral disease, malignancies other than breast cancer in the previous 3 years (except for adequately treated basal cell or squamous cell carcinoma of the skin or *in situ* carcinoma of the cervix), or a history of bleeding diathesis or long-term anticoagulant therapy. In addition, patients were excluded if they had received more than one regimen of chemotherapy or endocrine therapy for advanced disease, extensive radiation therapy or cytotoxic treatment in the previous 4 weeks, or strontium-90 in the previous 3 months. Patients with abnormal laboratory values were also excluded.

### Randomization and procedures

Eligible patients were randomized 1:1 to receive fulvestrant 500 mg or 250 mg, and given a sponsor-generated randomization code and corresponding patient pack. Randomization was stratified by prior endocrine therapy (antiestrogen or aromatase inhibitor) and enrollment of patients who had received prior aromatase inhibitor therapy was capped at 45% to ensure consistency with the international CONFIRM study (in which 42.5% of patients had received prior aromatase inhibitors) [11].

Patients in the fulvestrant 500 mg group received two 5 ml intramuscular (i.m.) injections of fulvestrant (one in each buttock) on day 0, 14, 28, and every 28 days thereafter. Patients in the fulvestrant 250 mg group received one 5 ml i.m. injection of fulvestrant and one placebo injection (one in each buttock) on day 1, 28, and every 28 days thereafter, and received two placebo injections on day 14. Treatment continued until disease progression or until any of the criteria for treatment discontinuation were met. Following database lock, patients were unblinded and transferred to open-label treatment, and patients in the fulvestrant 250 mg group no longer received placebo injections.

Patients had a clinical assessment once a month for the first 6 months of the study, and every 12 weeks thereafter. Disease progression was assessed by RECIST (version 1.1) every 12 weeks (± 2 weeks) from the first visit until progression. AEs were recorded at each study visit for the duration of the study and coded using the Medical Dictionary for Regulatory Activities (version 16.1).

### Endpoint measures

The primary endpoint of the study was PFS, defined as the time from the first study visit (randomization) to earliest objective disease progression, including death from any cause. Secondary endpoints included ORR (proportion of patients who had measurable disease at baseline and achieved a complete or partial response), DoR (time from response to progression or death in patients who had an objective response), CBR (proportion of patients in the full analysis set achieving a complete response, partial response, or stable disease ≥ 24 weeks), DoCB (time from randomization until disease progression or death from any cause in patients experiencing clinical benefit), safety, and pharmacokinetic data (to be reported separately).

### Statistical analysis

The study planned to randomize 220 patients in order to obtain at least 100 evaluable patients in each treatment group (the minimum number agreed with the China Food and Drug Administration for this registration trial). A predefined criterion was used to determine whether the results from this study were consistent with those from the international CONFIRM study; PFS was considered consistent if the HR for the treatment comparison (fulvestrant 500 mg vs 250 mg) was < 1 (i.e. favored fulvestrant 500 mg). The study was not powered to detect statistically significant differences between treatment groups.

The primary analysis for PFS was conducted using a log-rank test, stratified by prior endocrine therapy (antiestrogen vs aromatase inhibitor) in the full analysis set. The data cut-off and analysis was conducted when at least 150 progression events had been observed and the initial RECIST follow-up evaluation was completed for all randomized patients.

Subgroup analyses were performed using a log-rank test assessing PFS for six baseline covariates: age (< 65 vs ≥ 65 years), response to last endocrine therapy (responsive [recurrence after ≥ 2 years on adjuvant endocrine therapy, or complete response, partial response, or stable disease for ≥ 24 weeks on first-line endocrine therapy for advanced cancer] vs non-responsive), receptor status at diagnosis (both ER- and PgR-positive vs not both ER- and PgR-positive), visceral involvement (no vs yes), last endocrine therapy (antiestrogen vs aromatase inhibitor), and measurable disease (no vs yes).

The evaluable-for-response set (all randomized patients with at least one measurable target lesion at baseline) was used to determine the ORR and DoR. The full analysis set was used to determine the CBR and the DoCB. For ORR and CBR, a logistic regression model was fitted with factors of treatment and last endocrine therapy received prior to fulvestrant (antiestrogen vs aromatase inhibitor). DoR and DoCB were assessed by Kaplan-Meier analysis. Safety analyses were conducted for all patients who received at least one dose of fulvestrant.

## SUPPLEMENTARY MATERIALS FIGURE


