# Supplementary material for: Fulvestrant 500 mg vs 250 mg in postmenopausal women with estrogen receptor-positive advanced breast cancer: a randomized, double-blind registrational trial in China
Source: Oncotarget. 2016 Jun 23;7(35):57301–9. doi: 10.18632/oncotarget.10254 (PMC5302990; doi:10.18632/oncotarget.10254)
Supplement: Supplementary file 1 [file oncotarget-07-57301-s001.pdf]

Fulvestrant 500 mg vs 250 mg in postmenopausal women with estrogen receptor-positive advanced breast cancer: a randomized, double-blind registrational trial in China

Supplementary Materials

A

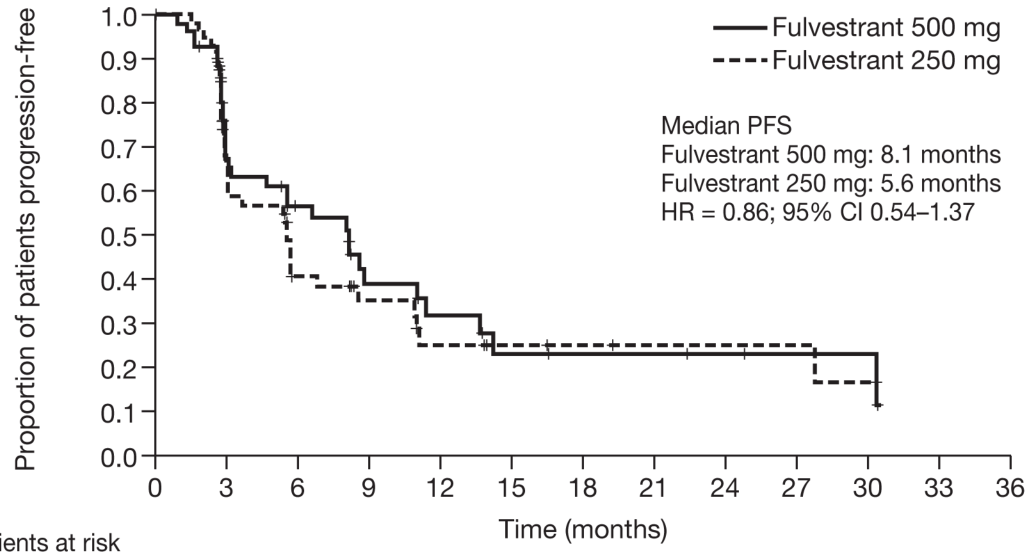

No. patients at risk												
Fulvestrant 500 mg	58	32	21	12	8	5	4	4	3	2	2	0
Fulvestrant 250 mg	63	33	18	11	7	5	4	3	3	3	2	0

B

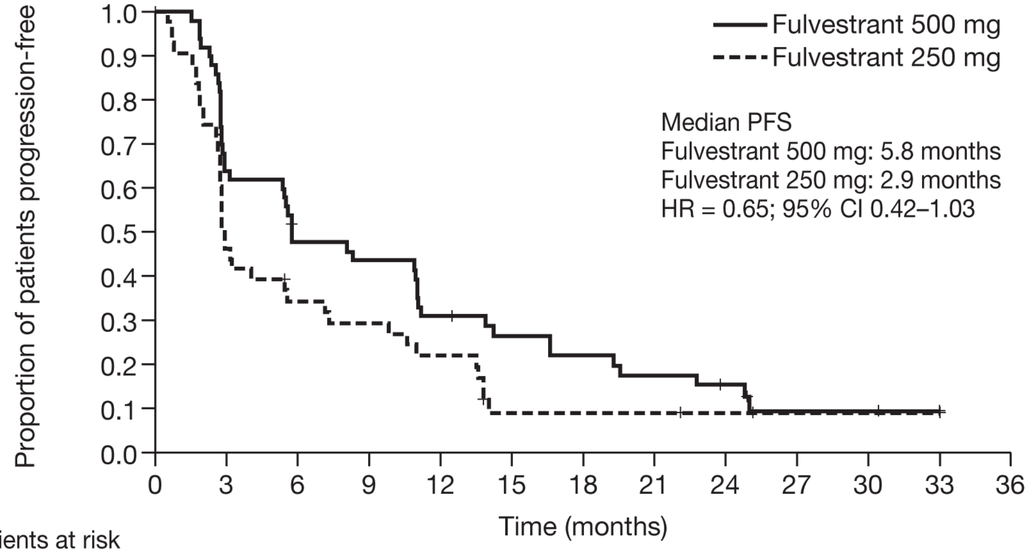

No. patients at risk													
Fulvestrant 500 mg	53	32	23	21	15	12	10	8	6	3	3	1	0
Fulvestrant 250 mg	47	20	14	12	9	3	3	3	2	1	1	1	0

Supplementary Figure S1: Kaplan-Meier analysis of PFS with fulvestrant 500 mg versus fulvestrant 250 mg in (A) the post-antiestrogen and (B) the post-aromatase inhibitor subgroups. CI, confidence interval; HR, hazard ratio; PFS, progression-free survival.
